# Grandmothers and Children’s Schooling in Sub-Saharan Africa

**DOI:** 10.1007/s12110-017-9306-y

**Published:** 2017-12-09

**Authors:** Sandor Schrijner, Jeroen Smits

**Affiliations:** Department of Economics, Institute for Management Research, Radboud University, PO Box 9108, 6500HK Nijmegen, The Netherlands

**Keywords:** Education, Family resource management, Grandchildren, Grandmothers, Poverty, Sub-Saharan Africa

## Abstract

Under poor circumstances, co-residence of a grandmother is generally considered to be beneficial for (grand)children. Empirical evidence does not unequivocally support this expectation and suggests that the grandmother’s importance depends on the family’s circumstances. We study the relationship between grandmother’s co-residence and children’s schooling in sub-Saharan Africa under a broad range of circumstances. Results make clear that the effect of a co-residing grandmother varies but is almost always positive. Grandmothers over age 60 are most effective in helping their (grand)children. They are particularly important for girls, and when the mother is deceased or not living in the household. Grandmothers are less effective in situations with few opportunities, as in very poor regions or in communities with few schooling opportunities. Our findings indicate that providing support to grandmothers should not be overlooked when designing policies aimed at strengthening the position of women and children in the sub-Saharan African context.

An important topic in family research concerns the benefits children in poor African countries derive from the presence of a grandmother in the household. The prevalent view that grandmothers are beneficial for their grandchildren is supported by much research (e.g., Gibson and Mace 2005; Hawkes et al. 1997; Hrdy 1999; Sear et al. 2000; for a broad overview see Sear and Mace 2008; Strassmann and Garrard 2011). However, there are also scholars who found negative or null effects of grandmothers on the well-being of their grandchildren (e.g., Borgerhoff Mulder 2007; Jamison et al. 2002; Strassmann 2011; Voland and Beise 2002). For example, Voland and Beise (2002) found that maternal grandmothers negatively affected child survival in historical Germany, and a recent study by Strassmann (2011) among the Dogon in Mali indicated that a co-residing grandmother may have adverse effects on survival and growth of their young grandchildren. Hence there seem to be circumstances in which grandmothers’ co-residence may have a negative impact on child well-being. This is a critical finding in light of the fact that in less-developed countries many children live in households with co-residing grandmothers, whether for cultural reasons or because of necessity (Kreidl and Hubatková 2014; Ruggles and Heggeness 2008). In designing policies aimed at improving the life chances of these children, it is important to find out under what circumstances co-residence of a grandmother has a positive effect on their grandchildren’s well-being. The current paper contributes to the field by providing new empirical evidence regarding the association between grandmothers’ co-residence and children’s schooling and how this association is moderated by the contexts in which the household is living.

Most research thus far consists of case studies focusing on one or a restricted number of groups or regions. These studies may provide in-depth understanding of the situation of those groups or regions, but they give less insight into the role of the households’ context. To study context in an effective (multivariate) way, information is needed on a large number of contexts and within each context on a large number of households. Such a multilevel database, with many households in many contexts, has not yet been used in research on the role of grandmothers in Africa.

Another limitation of the existing literature is that it is mostly focused on the relationship between grandmother’s co-residence and health outcomes, such as infant and child mortality and body growth (e.g., Borgerhoff Mulder 2007; Gibson and Mace 2005; Jamison et al. 2002; Sear et al. 2000; for a broad overview see Sear and Mace 2008; Strassmann and Garrard 2011). Research focusing on schooling is limited. Some studies examine the difference in school attendance between orphans and other children or the role of grandfathers in Africa, but in most of the research there is little attention for the role of grandmothers (e.g., Bicego et al. 2003; Hampshire et al. 2015; Kazeem and Jensen 2017; Nyambedha et al. 2003; Nyamukapa and Gregson 2005; Tamasane and Head 2010; see Parker and Short 2009 for an exception). This is regrettable because going to school is essential to increase the future earning opportunities of children. In sub-Saharan Africa, about 22% of the primary-school-age population still is not in school, and nonparticipation rates in secondary education are even (much) higher (UNESCO 2014). Gaining insight into the importance of a co-residing grandmother for children’s schooling and in particular the circumstances under which this role is most beneficial is therefore of great importance.

To study the relationship between grandmothers’ co-residence and children’s schooling in sub-Saharan Africa, we have built a new database with information on almost 900,000 children aged 7–15, living in 33 countries. By applying multilevel logistic regression analysis on this database, we aim to answer the following research questions:

1. What is the overall relationship between grandmother’s co-residence and their grandchildren’s educational participation in sub-Saharan Africa?
2. To what extent and in what way is this relationship influenced by situational factors, such as the age of the grandmother, resource- and gender-related characteristics of the household, and the context of the household?

Our approach takes a major step forward because we study the influence of context on the grandmother effect at the level of 1164 sub-national urban and rural regions—and for one factor even at the level of 29,925 communities—within 33 countries. This means that we have considerable power to study effects of context in a multivariate way and can answer questions about its role better than in earlier studies.

In the next section, we first discuss the importance of education and the reasons why grandmothers in sub-Saharan Africa may be living with their (grand)children. Then the theoretical framework that guides our research is presented and hypotheses are formulated. The third section describes the data and methods that are used. In the fourth section our results are presented, and in the final section we present our conclusions.

## Background

### Grandmothers and Schooling

Schooling influences future benefits by increasing human capital, which is crucial for economic development as well as for improving children’s prospects in life (Becker 1962). Although much research has already been done on the determinants of children’s schooling in poor countries (Glick and Sahn 2006; Huisman and Smits 2015; Lloyd and Blanc 1996; Mukherjee and Das 2008; Smits and Huisman 2013), the role of the grandmother has received relatively less attention. Only a few studies provide some evidence regarding this relationship. For example, Parker and Short (2009) found in Lesotho that living with a grandmother is beneficial for the children’s participation in education. Zeng and Xie (2014) showed for rural China that the educational level of coresident grandparents is positively associated with the educational attainment of their grandchildren. Kreidl and Hubatková (2014) found the negative effect of family size on reading test scores to be reduced in households with a co-residing grandparent, particularly at lower levels of economic development. Tamasane and Head (2010) found no difference in school attendance between South African children from single-parent households and children cared for by their grandparents.

There is also research regarding schooling outcomes that compares grandparent-headed households and households headed by other relatives. Children of grandparent-headed households in Malawi, Mozambique, and Zambia have better educational outcomes than those living in households headed by other relatives or nonrelatives (e.g., Ainsworth et al. 2005; Case et al. 2004; Nyamukapa and Gregson 2005). Regarding orphan status, a recent study by Kazeem and Jensen (2017) shows that Nigerian orphans have a higher chance of attending school if they are genetically more closely related to the household head. Several other studies investigate the difference in school attendance between orphans and other children (e.g., Bicego et al. 2003; Nyambedha et al. 2003; Nyamukapa and Gregson 2005), but in these studies little attention is paid to the role of grandmothers. Broad, comparative research that can show us how the relationship between coresident grandmothers and children’s schooling varies across circumstances is lacking for Africa, as well as for other low-income contexts.

### Why are Grandmothers Living with Their (Grand)Children?

To increase our understanding of the role played by co-residing grandmothers with respect to the schooling of their grandchildren, a first important question to be answered is why grandparents reside with their (grand)children.

In the African context, the reasons for this are diverse. In some regions and among some groups the cultural tradition exists that one or more children remain living with their parents after marriage (e.g., Fox 1967; Kandiyoti 1988; Korotayev 2003). The partners of these children then come to live in the family home and become a member of the extended family system. Especially under impoverished circumstances, sharing the costs of living increases survival chances and needs can be fulfilled more easily. Living together with their children also constitutes a natural old-age security system for the (grand)parents (Laferrère and Wolff 2006). Over time, the situation may gradually change from one in which the grandparents are the major driving forces of the household to one in which the next generation takes over. The grandparents then become the helping hands, as long as their health allows this.

Another way in which (grand)parents and children may come to live together is when married children establish their household elsewhere, but the grandparents move in later. This might be for financial reasons, because the grandparents need care, or because one of them has died. Depending on the (grand)parents’ health status, they may then be a resource or a burden for the household.

In both cases, when children after marriage remain living with their parents or when parents move in with their children, the outcome is a household in which grandparents are residing with their grandchildren in a three-generation setting. This three-generation household type should be distinguished from the skipped-generation household (Kreidl and Hubatková 2014) in which a fostering relationship between grandparents and grandchildren exists because parents are deceased or absent. Especially when mothers die, grandmothers are usually the ones that take over the care for their grandchildren. In sub-Saharan Africa, where overall mortality levels are high and an estimated 15 million children have lost one or both of their parents, this is a very common situation (Hampshire et al. 2015; Tamasane and Head 2010; UNAIDS 2013). Depending on the circumstances, the child may move to the household of the grandparent(s) or the grandparent(s) may come to live in the parental home.

## Theoretical Framework

### Grandmothers: A Drain or a Resource?

**Fig. 1:**
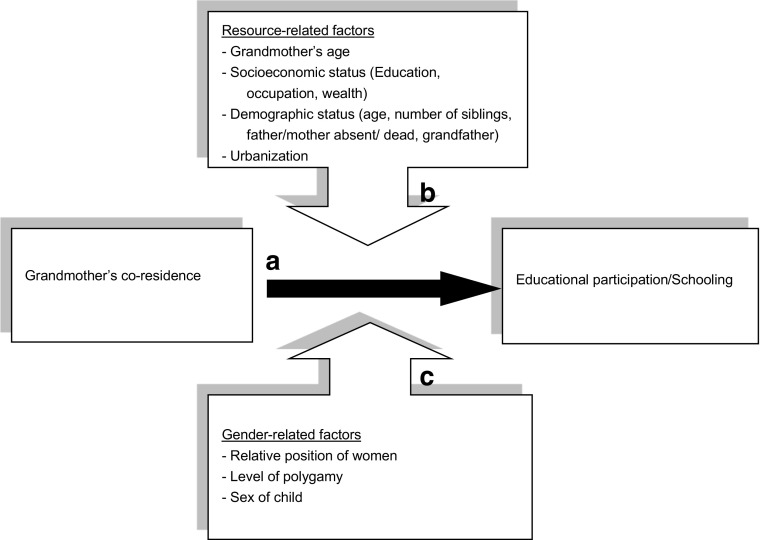
Conceptual model of relationship between grandmothers’ co-residence and children’s schooling in sub-Saharan Africa

The framework used in this study is presented in Fig. 1. Grandmothers’ co-residence is the major independent variable and educational participation the dependent variable. We hypothesize that the effect of grandmothers’ co-residence on their grandchildren’s schooling is positive (Arrow A in Fig. 1). This *grandmother co-residence hypothesis* is based on the expectation that grandmothers who are already living with their grandchildren have a low threshold to invest in their grandchildren. They are physically present in the household and consider it as their normal duty to contribute to the household and child rearing. Additionally, from a biological perspective, grandmothers may be predisposed to invest in their grandchildren. This idea is supported by evolutionary theory (e.g., Hawkes et al. 1997; Hrdy 1999, 2009; Sear 2008; Sear et al. 2000), whereby Hamilton's (1964) inclusive fitness rule plays a central role. According to this rule, individuals can enhance their inclusive fitness by reproducing themselves and/or by helping reproduce other kin with whom they share partly the same genes. As women age, the expected fitness returns from producing offspring themselves may be lower than the returns from helping rear their grandchildren and other kin. In line with this reasoning, the classical grandmother hypothesis argues that the healthy years a woman lives after menopause give her the opportunity to increase the reproductive success of her children. In this way, she also increases her own reproductive success (e.g., Hawkes 2004; Hawkes et al. 1997; Lahdenperä et al. 2004).

Grandmothers might also be a burden to the household resources. The *local resource competition hypothesis* (e.g., Borgerhoff Mulder 2007; Sear and Mace 2008) predicts that altruistic behaviour of family members may be reduced when local resources are scarce. Several studies support this hypothesis. Strassmann (2011) found the co-residence of a paternal grandmother led to a twofold higher hazard of death of a grandchild by the age of five. She attributes this to the fact that old grandmothers become net-consumers and therefore competitors with their grandchildren in the resource-poor society of the Dogon. Sear (2008) discovered that among the Chewa in Malawi child mortality rates are higher in the presence of matrilineal kin and in particular in the presence of the maternal grandmother. Sear supposed this negative effect to be caused by resource competition between kin. Borgerhoff Mulder (2007) observed, using within-population variation in land ownership in Kenya, that wealth affects the extent of kin altruism. Paternal relatives (specifically, father’s brothers) appear to buffer young children from mortality much more effectively in rich than in poor households. To what extent there is a positive grandmother effect on children’s schooling might thus depend on the circumstances, with the effect being weaker when the grandmother is old or the household is poor.

### Grandmothers and Child Survival

Many studies have analyzed the association between the presence of a grandmother and the physical well-being of their grandchildren. Hawkes et al. (1997) observed that Hadza grandmothers in Tanzania appear to enhance the nutritional welfare of their grandchildren by helping their daughters to obtain food for the children. In rural Gambia maternal grandmothers seem to double the survival chances of a Mandinka child by taking care of their grandchildren (Sear et al. 2000). For paternal grandmothers in that study, no effects were found. Ethiopian grandmothers had a positive effect on child survival by relieving their daughters of heavy domestic work. Non-reproductive maternal grandmothers in Ethiopia were positively associated with child height (Gibson and Mace 2005). However, when studying the Kipsigis in Kenya, Borgerhoff Mulder (2007) found no positive effect of maternal grandmothers, which she associated with the strong patrilineal organization of the Kipsigis. Strassmann (2011) also found no positive maternal grandmother effects. Dogon girls tend to grow faster in the absence of the maternal grandmother. According to Strassmann (2011:10899) this is probably the result of the hard work they have to perform for their grandmother, such as weeding the garden.

Studies in more affluent contemporary societies, such as the US and Europe, generally show a positive role of grandmothers (e.g., Danielsbacka et al. 2011; Dimova and Wolff 2010; Fuller-Thomson and Minkler 2001; Hank and Buber 2009; Kaptijn et al. 2013); for example, the grandmothers provided childcare or helped their (grand)children financially. For premodern societies, findings are more mixed (e.g., Beise 2005; Jamison et al. 2002; Lahdenperä et al. 2004; Voland and Beise 2002). Lahdenperä et al. (2004) found that the presence of grandmothers in eighteenth- and nineteenth-century Finland and Canada was associated with lowered mortality among their grandchildren, but that the significance of this effect depended on the grandmother’s and grandchild’s age. When a grandchild was between 2 and 5 years of age and grandmothers were under 60 at the children’s birth, survival probabilities were significantly higher. Using historical data (1671–1871) from a small village in Japan (Shumon Aratame Cho), Jamison et al. (2002) noted a positive association between the presence of a maternal grandmother and child survival rates. Presence of a paternal grandmother, on the other hand, was negatively associated with the survival of boys. Similar findings were obtained by Voland and Beise (2002), but in a very different historical context: the Krummhorn region in Germany in the period 1720–1874.

### The Role of the Circumstances

Given the variation in grandmother effects found in previous research, the main focus of the current paper is on the role of the circumstances; to what extent and in what way (negative or positive) a co-residing grandmother affects her (grand)children depends on her age and on specific characteristics of the household and the context (research question 2). This role of situational factors is represented in Fig. 1 by arrows B and C, which show the factors that moderate the grandmother effect (Arrow A). Situational factors may be resource-related or gender-related and are located at the household and context level. In the next section, these factors and other control variables are discussed.

#### Grandmother’s Age

As discussed above, the local resource competition hypothesis (Borgerhoff Mulder 2007; Sear and Mace 2008) emphasizes the negative effects on altruistic behavior of family members due to scarcity of local resources. In a resource-poor environment, a grandmother may easily become a competitor with her grandchildren, especially when she is old and needs to be taken care of (cf. Strassmann 2011). There is also evidence that the survival probabilities of children in eighteenth- and nineteenth-century Finland and Canada were significantly higher when their grandmothers were under 60 at their birth (Lahdenperä et al. 2004).

However, the grandmother should also not be too young. If grandmothers are still reproductive themselves, they have to put their energy into caring for their own offspring and have less opportunities to take care of their grandchildren. Sear et al. (2000) found evidence in rural Gambia that young children living with non-reproductive grandmothers are taller than those living with grandmothers who are still reproductively active, and Hawkes et al. (1997) report that nonreproductive Hadza grandmothers put much more effort into the acquisition of food than reproductive women/grandmothers do.

Hence, regarding the relationship between the age of the grandmother and her importance as a positive resource for her grandchildren we would hypothesize a nonlinear (parabolic) relationship, with her contribution being highest in the middle age range (no young children of her own and not yet too old to contribute). This *parabolic age effect hypothesis* will be tested by looking at nonlinear effects of grandmother’s age in our analysis.

#### Resource-Related Factors

Resource-related factors at the level of the household are education, employment, and income/wealth, There is broad evidence that children with less-educated parents or whose fathers have a farm job go to school less often and have higher dropout rates (Buchmann and Brakewood 2000; Colclough et al. 2000; Ersado 2005; Huisman and Smits 2015; Mingat 2006; Smits and Gündüz-Hoşgör 2006). Better-educated parents (have) experience(d) the benefits of education themselves and therefore are expected to weigh the costs and benefits more in favor of schooling than parents with little education (Huisman and Smits 2009; Piotrowski and Paat 2012). Fathers working in the agricultural sector are expected—and have been found (Breen and Goldthorpe 1997; Huisman and Smits 2015)—to attach less value to schooling than those who work in other occupations. Regarding income and wealth, we know that children of poor families are less often enrolled in school, are more involved in child labor, and suffer from many other negative outcomes, including high levels of child mortality, disease, and stunting (Basu and Tzannatos 2003; Bourdillon 2006; Duncan and Brooks-Gunn 1997; Hope 2005; Webbink et al. 2012). The direct and indirect costs of schooling are a heavy economic burden for many households (Admassie 2003; Ananga 2011; Lloyd and Blanc 1996). Under such difficult circumstances, a co-residing grandmother can play an important role for children’s schooling, even though many African grandmothers have little education themselves. A grandmother in a three-generation household can enable parents to work outside the home, prevent children from having to take over household tasks, free them from working in a family business, and provide them with encouragement and emotional support (Kreidl and Hubatková 2014; Levetan and Wild 2016).

The presence of a grandmother is expected to be particularly important in skipped-generation households, when parents are deceased or absent from the household and grandparents and children are in a fostering relationship. Parental death and especially maternal death is known to have a negative impact on children’s well-being and schooling outcomes (e.g., Case and Ardington 2006; Evans and Miguel 2007; Nyamukapa and Gregson 2005). Also single parenthood is associated with negative effects on children’s schooling (Martin 2012; Pong and Ju 2000; Potter 2010). It seems likely that under these circumstances, living with a grandmother may be particularly beneficial to children’s well-being and schooling. Research by Parker and Short (2009) in Lesotho and Kazeem and Jensen (2017) in Nigeria confirms this for the African context.

Important resources in the local context are the educational and transport infrastructure, which both may influence the likelihood that children will go to school. In sub-Saharan Africa, the availability of (good quality) schools and infrastructure varies considerably according to the overall level of urbanization and development of the region. In more modern and urban areas, infrastructure is generally better and state influence stronger, which means that educational laws may be better enforced. The effects of globalization may also be stronger, and value patterns that stress the importance of education and equality between the sexes more widespread. This might put pressure on parents to send their children to school (Huisman and Smits 2009; Tansel 2002). Smits and Gündüz-Hoşgör (2006) found for Turkey that children in urban areas have significantly higher schooling attainments, and Fafchamps and Wahba (2006) found for Nepal that children living near towns and cities are more likely to attend school. Hence, the expectation is that particularly in rural areas a co-residing grandmother might increase young children’s chances to go to school.

#### Gender-Related Factors

Most of the studies examining the relationship between the presence of a grandmother and the well-being of grandchildren report different outcomes for boys and girls (e.g., Borgerhoff Mulder 2007; Gibson and Mace 2005; Jamison et al. 2002; Strassmann 2011). Hence gender-related factors should be included in our analysis as well.

In most regions of sub-Saharan Africa, women are traditionally responsible for the day-to-day care of children and to a large extent for their economic support (Caldwell and Caldwell 1987; Kandiyoti 1988). There is evidence that women having a stronger position in society is associated with children having better education, health, and well-being (e.g., Hobcraft 1993; Mukherjee and Das 2008). Given that in regions where the position of women is stronger the position of grandmothers also tends to be stronger, the expectation is that in such regions the presence of a grandmother is particularly beneficial.

In addition to the general position of women, the presence and extent of polygamy might be important too. Strassmann (2011:1) observed that child mortality and stunting rates are significantly higher in polygamous families. She attributed this to the fact that polygamy creates conflicts within families associated with asymmetries in genetic relatedness. If there is more uncertainty about genetic relatedness among family members, the risk of conflict increases. Omariba and Boyle (2007) found that children from polygamous families have higher mortality rates than those from monogamous families. Kandiyoti (1988:277) argues that in case of polygamy the continuing obligations of both men and women to their own kin do not foster a notion of the family or household as a corporate entity. To what extent this is also true for grandmothers living in these families is not clear. Hence, whether the effect on schooling of the presence of a grandmother in such families is stronger or weaker than in a monogamous family remains an empirical question to be answered in our analyses.

#### Other Factors

Other factors that may affect the grandmother co-residence effect are the number of children and the birth order of a child. Regarding the number of children, literature indicates that the probability of going to school is smaller for children with more siblings (Booth and Kee 2009; Huisman and Smits 2009). A likely explanation is that children with more brothers and sisters have to share the available resources. With regard to birth order there is evidence that older children, in particular older girls, have lower schooling rates, probably because they have to work in the household or earn money to supplement household income (Buchmann and Hannum 2001; Emerson and Souza 2008; Webbink et al. 2013). In both cases, it seems likely that the presence of a grandmother may help overcome negative situations. We therefore expect the presence of a grandmother in the household to be more important in high-fertility situations and for elder daughters.

## Data and Methods

### Data

For this study, combined datasets from the sub-Saharan African Demographic and Health Surveys were used (DHS; www.dhsprogram.com). The data are derived from the Database Developing World of the Global Data Lab (www.globaldatalab.org). DHS are large, nationally representative household surveys. For each survey, non-overlapping areas (often enumeration areas) are randomly selected. These areas (called “clusters” henceforth) are usually communities, villages, or city quarters. In the selected clusters, all households are listed and a random sample of 25–30 households is selected for the interviews. The DHS consists of a household survey, in which basic information is collected of all household members, and separate women’s and men’s surveys. In the women’s surveys, all usual resident women aged 15 to 49 are invited for an oral interview. In this interview, information is obtained on socioeconomic, demographic, and health-related issues.

To maximize discriminatory power, the data from all available standard DHS surveys for sub-Saharan African countries held since 2000 have been pooled. For South Africa and Togo data from 1998 are used because at the start of our research no other DHS surveys for these countries were available. To control for the fact that the surveys are held in different years and that for most countries several surveys were brought together, an indicator for survey year is included in the analysis. Appendix Table 3 provides additional information about the sample. Response rates are generally very high: >95% in all but one survey.

Our combined dataset contains information derived from 69 surveys on 917,788 children (467,528 boys and 450,260 girls) aged 7–15 living in 29,925 communities (sample clusters) within 1164 urban and rural sub-national regions (called “districts” henceforth) of 33 sub-Saharan African countries. The household-level data has been supplemented with context information at the level of districts and communities/clusters. To get representative samples of the countries, the household weights provided by DHS are used in all analyses. Because of cases with missing data on parental education, (grand)parental age, polygamy, number of brothers and sisters, wealth, and educational participation, and some unrealistic cases for (grand)parental age, 19,782 (2.2%) children have been removed from the dataset. Unrealistic cases are parents younger than 19 or grandmothers under 31 (since the children are at least 7). Our analysis therefore covers 898,006 children (457,286 boys and 440,720 girls). Missing characteristics of parents and grandmothers who were absent from the household (e.g., education or occupation of a deceased father) are addressed using the dummy variable adjustment procedure, which leads to unbiased estimates of these variables (Allison 2001; Little and Rubin 2002).

### Method and Variables

The dataset is hierarchical. Households are nested within sample clusters nested within districts nested within countries. We use three-level logistic regression analysis to address the nesting of the households within sample clusters and districts and include fixed effects dummies at the national level to control for the nesting within countries. This strategy allows us to control for clustering and confounding at the national level while retaining the possibility to study the role of context factors at the district and cluster level.

The dependent variable “educational participation” is a dummy variable indicating whether (1) or not (0) children aged 7–15 were attending school at the time of the interview. The upper age limit of 15 is chosen because above that age fewer children are living in their natal household (e.g., because of early marriage, education, or orphanhood). The lower age limit is set at 7 because in most sub-Saharan African countries a substantial number of children do not start schooling until after the compulsory age (Huisman and Smits 2009). The models are estimated with MLwiN, using second-order penalized quasi-likelihood (PQL2), the recommended estimating technique for multilevel logistic regression analysis (Goldstein and Rasbash 1996).

The major independent variable is a dummy variable indicating whether (1) or not (0) children are living with a grandmother. Children co-resident with their grandmother are identified in the DHS data by using the household roster, which defines for all household members the relationship to the household head. Children are identified as living with a grandmother if (1) they are grandchildren of a female household head; (2) they are grandchildren of a male household head whose wife is also living in the household; (3) they are children of the household head, and the mother or mother-in-law of the household head is also living in the household; (4) they are children of a brother or sister of the household head, and the mother of the household head is also living in the household. All other children, including adopted and foster children, are considered as not living with their grandmother. Given the limited information on the relationships within the households, it cannot be completely precluded that some of these children still live with a grandmother—for example, if they belong to the categories “Other family members” or “Not related household members.” However, given that the number of school-aged children in the data who belong to these categories is very small (3%), the number of them living with a grandmother is expected to be negligible.

In situation 2 the grandmother might not be the biological grandmother of the child because of polygamy. For polygamous households, there is insufficient information to determine which of the household head’s wives is the “real” grandmother. To control for this situation, a dummy variable has been added to the models, indicating whether (1) or not (0) the household is polygamous (the head has more than one wife). To find out whether this situation influenced the grandmother effect, the presence of an interaction of this variable with the grandmother dummy has been tested. This interaction was not significant.

Other independent variables are the grandmother’s age, measured in years, and resource- and gender-related factors at the household and context level. The presence of each parent is measured with two dummies, one indicating whether (1) or not (0) the parent is absent from the household and one indicating whether (1) or not (0) the parent is deceased. Age of the child and age of its (grand)mother are interval variables. The variables “number of sisters” and “number of brothers” are interval variables ranging from 0 to 10 or more. Birth order is an interval variable ranging from 0 to 18 or more.

The models contain a number of control factors that are known or can be expected to influence children’s educational participation. Household wealth, father’s occupation, parental education, employment of the mother, and the presence of a grandfather in the household are factors that have been known to influence children’s educational participation (Evangelista de Carvalho Filho 2012; Glewwe and Jacoby 2004; Mingat 2006; Schrijner and Smits 2017; Shavit and Blossfeld 1993; Smits and Gündüz-Hoşgör 2006).

Because income is lacking in the DHS data, household wealth was measured by the International Wealth Index (IWI; Smits and Steendijk 2015), a comparative asset-based wealth index. IWI indicates to what extent the household owns a basic set of assets, valued highly by people across the globe, such as TVs, cars, telephones, and housing characteristics such as the quality of the floor material and toilet facility. Education of the mother and father is measured in years of education completed. Occupation of the father is measured by three dummy variables indicating whether (1) or not (0) the father was employed on a farm or in other lower (sales, services, manual) or upper (professional, technical, managerial, clerical) occupations. Employment of the mother is measured by a dummy variable indicating whether (1) or not (2) the mother aside from her housework did any other work last week. The questions used in the DHS surveys for measuring women’s employment are: “Aside from your own housework, have you done any work in the last seven days?” And if the answer was no: “As you know, some women take up jobs for which they are paid in cash or kind. Others sell things, have a small business or work on the family farm or in the family business. In the last seven days, have you done any of these things or any other work?” Women who answered yes on one of these questions are considered as being employed. To indicate the relative position of the mother in the household we follow earlier research (Blanc and Wolff 2001; Luz and Agadjanian 2015; Spierings et al. 2010) and use the age difference between the parents (age of mother minus age of father).

To study the importance of context factors, socioeconomic characteristics (level of development, urbanization, education) and gender-related cultural characteristics (age difference between spouses, polygamy) of the region have been added to the models. Level of development is indicated by the mean of the International Wealth Index in the region. Given that this index at the national level is highly correlated with the Human Development Index and with GNP per capita (Smits and Steendijk 2015), it is expected to be a good development indicator at the sub-national level as well. Urbanization is measured by a dummy variable indicating whether (1) or not (0) the household is in a rural area. Education is measured by the mean years of education of people aged 20–40 in the area. The relative position of women in the household’s context is indicated by the average age difference between parents as an interval variable and by the percentage of polygamous households in the area. Polygamous households are households where the male household head has more than one wife.

Given that for African countries hardly any indicator is available at the sub-national level, context factors are created by aggregating household level variables to the sample cluster and district level. Sample clusters are villages or neighborhoods and therefore accurately reflect the nearby community. Using context variables at the cluster level seems preferable over using such variables at the more distant district level. However, the sample clusters in our data are rather small (at most 30 households and often much less). This means that there is little variation at that level and measurement is imprecise. At the district level, sample sizes are much larger. There is also evidence that context effects can be caught rather well by more distant variables (Smits et al. 2005). To find out which level would be best for each of the context factors, they have been tested at both levels. It turned out that education had its strongest effect at the cluster level (in line with earlier findings of Kravdal 2006), but that wealth, age difference, and polygamy had their strongest effect at the district level. We therefore include context education at cluster level and the other context factors at district level.

To find out whether and in which ways the effect of a co-residing grandmother differs across circumstances, we have tested for interactions between the grandmother dummy and other variables at the household and context levels and added the significant interactions to the model. In this interaction analysis, centered versions of the involved variables are used so the main effects can be interpreted as average effects.

## Results

Table 1 provides descriptive statistics. We observe that 16% of the children aged 7–15 in our sample are living with at least one grandmother and 73% are attending school at the time of interview. The average age of grandmothers in the sample was nearly 63 years. For 35% of the children the father is absent or deceased, and for 23% of the children the mother is absent or deceased. Almost 71% of the children are living in a rural area.

**Table 1:** Descriptive statistics: Percentages, means of characteristics of children aged 7–15

Variables	%, mean	Min	Max	SD
School attendance (dependent variable)	73.0%	0	1	0.44
*Household factors*				
Grandmother in household	16.2%	0	1	0.37
Grandfather in household	6.8%	0	1	0.25
Age of grandmother	62.9	31	98	4.37
Sex of child is girl	49.1%	0	15	0.50
Age of child	10.7	7	15	2.54
Age of mother	38.0	19	98	7.39
Age of father	46.9	19	98	8.80
Birth order	3.30	1	18	1.94
Number of sisters	1.92	0	10	1.65
Number of brothers	2.04	0	10	1.74
Mother alive, not in household	19.0%	0	1	0.39
Mother deceased	4.4%	0	1	0.20
Father alive, not in household	25.9%	0	1	0.44
Father deceased	9.6%	0	1	0.29
Household wealth (IWI)	27.0	0	100	22.73
Education of father (years)	4.13	0	16	3.79
Education of mother (years)	2.99	0	16	3.47
Mother employed	69.3%	0	1	0.46
Father’s occupation:				
Farm (reference category)	60.4%	0	1	0.49
Lower nonfarm	29.5%	0	1	0.46
Upper nonfarm	10.1%	0	1	0.30
Position of mother (age of mother − age of father)	−9.39	−73	60	7.79
Polygamous household	12.9%	0	1	0.33
*Context factors*				
Living in rural area	70.7%	0	1	0.46
Level of development (district)	27.02	0.99	88.96	16.93
Educational level (cluster)	2.9	0	12.5	1.30
Position of women (district)	−8.99	−27.1	0.04	2.64
Polygamy (district)	29.0%	0	1	0.19

Source: 1998–2013 DHS (www.dhsprogram.com)

Table 2 shows the results of the multilevel logistic regression analyses. Regarding the educational participation of the children, the models perform in line with what is already known (e.g., Huisman and Smits 2009, 2015). Children are more often in school when their parents are more highly educated, the father is a a nonfarm worker, the mother is employed, the household is wealthier, there are fewer siblings in the household, there is a more traditional situation with regard to the age difference between the parents (father older than mother) and the mother is not absent or deceased. They also more often are in school when they live in an urban area and in one with a higher educational level. Children are less often in school when they are living in a polygamous environment. District level of development has a negative sign, which is unexpected. This is probably due to some multicollinearity with household level of development, as both are based on the same wealth index. Indeed, when household level wealth is removed from the models, the coefficient of district level of development becomes positive. This multicollinearity is not problematic for our outcomes regarding the grandmother effect because it is between two control factors in the models (Allison 2012; Voss 2004). Removing either or both wealth-based variables from the models does not affect these outcomes at all. Given that the models perform well in all other respects, we accept them as good models for studying the effect of grandmother’s co-residence on children’s schooling.

**Table 2:** Multilevel logistic regression analyses of the school attendance of children aged 7–15 in 33 sub-Saharan African countries: log odds, standard errors and odds ratios†

	Model 1			Model 2		
	β	SE	Exp(β)	β	SE	Exp(β)
*Fixed part*						
Intercept	1.35***	0.156		1.39***	0.128	
Grandmother (Gm) in household	0.355***	0.018	1.43	0.354***	0.024	1.43
Gm age	0.033***	0.008	1.03	0.033***	0.008	1.03
Gm age^2^	−0.0003***	0.000	1.00	−0.0003***	0.000	1.00
*Household factors*						
Age child	0.029***	0.007	1.03	0.029***	0.007	1.03
Sex of child is girl	−0.251***	0.021	0.78	−0.249***	0.020	0.78
Age mother	0.038***	0.005	1.04	0.036***	0.004	1.04
Birth order child	−0.023***	0.003	0.98	−0.024***	0.003	0.98
Number of sisters	0.007*	0.003	1.01	0.009**	0.003	1.01
Number of brothers	−0.027***	0.003	0.97	−0.025***	0.003	0.98
Mother not in household	−0.527**	0.166	0.59	−0.521**	0.165	0.59
Mother deceased	−0.599***	0.166	0.55	−0.565***	0.165	0.57
Father not in household	−0.314	0.167	0.73	−0.322	0.166	0.72
Father deceased	−0.344*	0.166	0.71	−0.353*	0.166	0.70
Grandfather in household	−0.025	0.019	0.97	0.083*	0.035	1.09
Household wealth (IWI)	0.027***	0.000	1.03	0.027***	0.001	1.03
Education father (years)	0.079***	0.002	1.08	0.078***	0.002	1.08
Education mother (years)	0.086***	0.003	1.09	0.087***	0.003	1.09
Mother employed	0.145***	0.015	1.16	0.144***	0.015	1.15
Occupation father (ref = farm)						
Lower nonfarm	0.113***	0.024	1.12	0.086***	0.025	1.09
Upper nonfarm	0.223***	0.048	1.25	0.250***	0.052	1.28
Position mother (mother’s − father’s age)	0.003***	0.001	1.00	0.002***	0.001	1.00
Polygamous household	−0.115***	0.015	0.89	−0.120***	0.015	0.89
*Context factors*						
Living in rural area	−0.530***	0.081	0.59	−0.504***	0.082	0.60
Level of development (district)	−0.014***	0.003	0.99	−0.013***	0.003	0.99
Educational level (cluster)	0.150***	0.014	1.16	0.151***	0.014	1.16
Position women (district)	0.026	0.021	1.03	0.024	0.021	1.02
Polygamy (district)	−1.66***	0.301	0.19	−1.73***	0.304	0.18
Year	0.048***	0.007	1.05	0.049***	0.007	1.05
*Interactions with grandmother (Gm) dummy*						
Gm × Sex is girl				0.117***	0.020	1.12
Gm × Mother not in household				0.545***	0.035	1.72
Gm × Mother deceased				0.449***	0.047	1.57
Gm × Number of sisters				−0.021**	0.006	0.97
Gm × Occupation father (lower nonfarm)				−0.272***	0.080	0.76
Gm × Grandfather in household				−0.235***	0.047	0.79
Gm × Educational level (cluster)				0.039***	0.011	1.04
Gm × Level of development (district)				0.007***	0.001	1.01
Gm × Position mother				−0.007***	0.002	0.99
Gm × Living in rural area				0.172***	0.042	1.19
*Random part (random slopes model)*						
District level (3)						
Variance intercept schooling	0.452***	0.027		0.450***	0.027	
Variance grandmother random slope	0.051***	0.007		0.032***	0.006	
Cluster level (2)						
Variance intercept schooling	0.764***	0.031		0.765***	0.031	
Variance grandmother random slope	0.433***	0.020		0.426***	0.020	

***p < 0.001

**p < 0.01

*p < 0.05 (*N* = 898,006 of whom 145,444 are living with a grandmother and 655,783 are attending school)

†Both models include the full set of country-level fixed effects dummies to control for confounding and clustering at the national level. The coefficients of the fixed effects dummies are presented in Appendix Table 4

### The Grandmother Effect

**Fig. 2:**
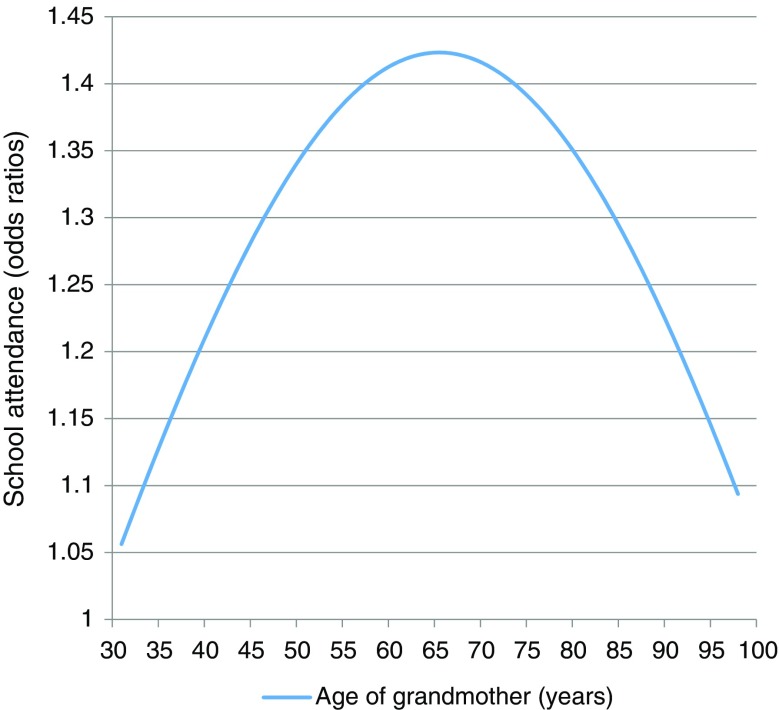
Predicted relationship between age of co-residing grandmother and the odds ratios of being in school based on the grandmother’s age and age^2^ variables from model 2 (Table 2)

Model 1 shows that the co-residence of a grandmother is positively associated with the educational participation of their grandchildren. This effect is significant and substantial. When controlling for the other major risk factors at the household and context level, the odds of being in school are about 40% higher for children living with a grandmother. This finding is in line with the broadly held view that the presence of a grandmother is beneficial to their grandchildren. We also observe that the strength of the grandmother effect depends nonlinearly on her age. If the grandmother is young or old, the effect is weaker than if she is in her middle age. The shape of the relationship is displayed in Fig. 2, which presents the odds ratios of the combined effect of the linear and quadratic coefficients of grandmother’s age. We see that the grandmother effect is strongest for grandmothers in their late sixties and that for young or very old grandmothers it is weaker. Children living with a grandmother aged 66 have 18% higher odds of being in school than children with a grandmother aged 40. The findings are in line with the ideas that young grandmothers may be less of a resource because they are reproductive themselves and that (very) old grandmothers may be more of a burden than a resource for the household. They also suggest that African grandmothers in their sixties and seventies are not too old to provide a substantial contribution to the household.

### The Role of Context

The random effect variances related to the co-residence of a grandmother are presented at the bottom of Table 2. These variances are highly significant and sizable, indicating that the grandmother effect varies considerably across districts and clusters. Hence, it makes sense to study interactions between grandmother’s co-residence and the district- and cluster-level variables. The results of the interaction analysis are presented in Model 2 (Table 2). The number of significant interactions between the presence of the grandmother and other variables at the household, cluster, and district levels is sizeable. A first important interaction is with the gender of the grandchild: co-residence of a grandmother is significantly more important for girls than for boys. For girls, the effect of a co-residing grandmother is 12% higher than for boys.

Presence of a grandmother is particularly important if the mother is deceased or absent from the household. In those situations, the size of the grandmother effect is increased by 57% and 72%, respectively. In case of a deceased or absent father, presence of a grandmother does not make a significant difference for children’s schooling. Hence it seems that grandmothers can replace a missing mother, but less so a missing father. The negative coefficient of the interaction with the number of sisters might indicate that children’s schooling is hindered by household tasks that are either performed by the grandmother or by co-residing (probably older) sisters. When the father is employed in a lower nonfarm job, the grandmother effect is 24% lower than when he is farmer. Hence, grandmothers are more important in farm households than among nonfarm manual workers. Grandmothers are also more important when there is no grandfather in the household.

In addition, there are also significant interactions with characteristics at the level of context. Both education (at cluster level) and level of development (at district level) show positive and significant interactions with the grandmother variable. This suggests that presence of a grandmother is more favorable for children’s schooling in situations where schooling opportunities are already rather good. The effects are also quite strong. For each one-year increase in the educational level of the local community, the grandmother effect is 4% stronger. And for each step increase in the district on the international wealth index, the grandmother effect is 1% stronger. Given that the local educational level varies between 0 and 12 years of education and that district level of development varies between 1 and 89 on the international wealth index scale, these differences are substantial.

Table 2 also shows that grandmothers are more important in rural areas. In those areas, the grandmother effect is 19% stronger than in urban areas. This result is in line with the finding discussed above, that grandmothers are more important in farm households than in households where the father works in a lower nonfarm occupation. An explanation might be found in the fact that most of the grandmothers in our data grew up in rural areas. Those who are still living there probably have more possibilities to contribute to the household than those who moved to the city, where the environment is less familiar to them and they more easily become a burden to the household. We also see that in households where the mother's position is stronger, less support from a grandmother is needed.

The grandmother effect does not differ between polygamous and non-polygamous households, nor according to the level of polygamy in the region. It is also not related to survey year, which implies that the importance of a co-residing grandmother has been more or less stable over time in the period studied.

## Conclusion and Discussion

On the basis of data on almost 900,000 children aged 7–15 and living in 33 sub-Saharan African countries, we find broad evidence that living with a grandmother is positively associated with the likelihood that young children will be in school. Although the interaction analysis shows that certain conditions may weaken the grandmother effect, no indications of negative grandmother effects were found. We therefore can conclude that—at least for the educational participation of children—the presence of a grandmother in the household is a positive resource under a broad range of circumstances within the sub-Saharan African context. Hence grandmothers are not only important for the life chances of very young children, as was already known from earlier research (e.g., Hawkes et al. 1997; Hrdy 1999; Kreidl and Hubatková 2014; Sear and Mace 2008; Sear et al. 2000), but also for those of older, school-age children.

Even though many of these grandmothers have little education themselves, they can contribute to the schooling of their grandchildren in several ways. They can compensate the (direct and indirect) costs of schooling, which are a heavy economic burden for many African households (Admassie 2003; Ananga 2011; Lloyd and Blanc 1996). In a three-generation household, they can enable parents to work outside the home, prevent children from having to take over household tasks, free them from working in a family business, and provide them with encouragement and emotional support (Kreidl and Hubatková 2014; Levetan and Wild 2016). In a skipped-generation household, in which parents are deceased or absent, their role can be even more important. Parental death and especially maternal death is known to have a negative impact on children’s well-being and schooling outcomes (e.g., Case and Ardington 2006; Evans and Miguel 2007; Nyamukapa and Gregson 2005) and also absence of parents due to divorce or separation may profoundly affect children’s lives (Amato 2000; Chae 2016; Pong et al. 2003). Our results make clear that presence of the grandmother is indeed particularly important when the mother is deceased or absent from the household. However, absence or death of the father does not influence the grandmother effect at all. Hence grandmothers may replace a missing mother, but less so a missing father. The presence of a grandfather in the household is negatively related to the grandmother effect, which seems to suggest that when her husband is present the grandmother’s resources are more directed toward him than toward the grandchildren.

Girls profit more from a co-residing grandmother than boys. This indicates that grandmothers take over household tasks that otherwise would have been done by girls. The extent to which grandmothers contribute to children’s schooling also depends on grandmother’s age in a nonlinear way (Fig. 2): The contribution is highest for grandmothers who are in their sixties or seventies and lower for grandmothers who are younger or older. This is not surprising since young grandmothers may still have children at home to care for. Very old grannies may need care themselves and become net consumers, competing for resources with other family members in the household, especially under poor circumstances.

Interestingly, a co-residing grandmother is particularly good for children’s schooling when the household lives in a more developed (wealthier) environment or in a community with a more highly educated population. It seems that grandmothers help the family to make better use of available favorable circumstances. This result is in line with the local resource competition hypothesis, which predicts altruistic behavior of family members to be reduced when there is scarcity of local resources (and thus to be enhanced under more favorable circumstances). However, this result is only found for resources in the context in which the household lives. Wealth and education at the household level are not related to the grandmother effect; poor and uneducated households profit as much from a co-residing grandmother as wealthier and educated households. Also the work status of the mother makes no difference, though the occupation of the father does. Grandmothers are more important when the father is employed in a farm job instead of another lower-paying labor job. A possible explanation may be that most of the grandmothers in our data grew up in rural areas, often at farms. This means that they are probably more familiar with farm life—and can contribute more to the household there—than in a nonfarm environment. The fact that the grandmother effect is weaker in urban areas also points in this direction.

No significant interaction is found with age of the children, which indicates that grandmothers are equally important for children at the primary and the lower secondary school level. In households where the position of the mother is stronger, as measured by the age difference with her husband, and in households with more (grand) daughters, a co-residing grandmother makes less of a difference. If mothers have stronger bargaining power, or if household tasks can be divided among more daughters, there might be less need for a grandmother to contribute to the household. Given the negative effects of polygamy on child survival documented in earlier research (e.g., Omariba and Boyle 2007; Strassmann 2011), we were wondering whether the grandmother effect on children’s schooling would be affected by polygamy, at the household or community level. This turned out not to be the case. The contribution of a grandmother in these households and communities seems to be of a general nature that benefits all children to more or less the same extent.

Some caution is required regarding our conclusions, as our study has several limitations. First, it is based on cross-sectional data. Although interesting new information is obtained on the association between grandmothers’ co-residence and children’s schooling and on the variation of this relationship across circumstances, no strict conclusions in terms of causal relations can be drawn. Second, as our data does not contain information on non-residing grandmothers, it is not possible to say anything about the distance gradient in grandmother support. Grandmothers who live in the vicinity of their (grand)children are probably better able to support them than grandmothers who live farther away. Insight into the nature of this relationship is important for policy makers and social agents who want to strengthen existing family ties in order to improve the position of children. Further research is therefore needed on this distance gradient, as well as on other important factors, such as the role played by local organizations, schools, governmental services, and NGOs.

In sum, we found evidence of a positive grandmother effect on children’s schooling across a broad range of circumstances in the sub-Saharan African context. Compared with earlier research, our study is a major step forward because it provides—for the first time—a broad comparative analysis of the role played by context in the relationship between grandmother’s co-residence and child well-being—in particular, children’s schooling. Our findings clearly indicate that grandmothers should not be overlooked when designing policies aimed at strengthening the position of women and children in the sub-Saharan African context.

## Appendix

**Table 3:** DHS country data, year of survey(s) and household response rates

Country	Year(s)	HH Resp. rate (%)
Benin	2001, 2006, 2011	97.0, 99.1, 98.6
Burkina Faso	2003, 2010	99.4, 99.2
Burundi	2010	99.1
Cameroon	2004, 2011	97.6, 99.0
Chad	2004	99.4
Cote d’Ivoire	2005, 2011	95.5, 98.1
Congo DR	2007, 2013	99.3, 99.9
Congo Brazzaville	2005, 2011	99.2, 99.8
Ethiopia	2000, 2005, 2011	99.3, 98.5, 98.1
Gabon	2000, 2012	97.6, 99.3
Ghana	2003, 2008	98.7, 98.9
Guinea	2005, 2012	99.2, 99.5
Kenya	2003, 2008	96.3, 97.7
Lesotho	2004, 2010	95.2, 97.6
Liberia	2007, 2013	97.2, 99.4
Madagascar	2004, 2009	97.8, 98.8
Malawi	2000, 2004, 2010	99.0, 97.8, 98.1
Mali	2001, 2006, 2013	97.9, 98.8, 98.4
Mauritania	2001	98.4
Mozambique	2003, 2011	80.6, 99.8
Namibia	2000, 2006, 2013	96.9, 97.8, 96.9
Niger	2006, 2012	98.0, 98.0
Nigeria	2003, 2008, 2013	98.6, 98.3, 99.0
Rwanda	2000, 2005, 2010	99.7, 99.7, 99.8
Senegal	2005, 2011, 2012	98.5, 98.4, 98.7
Sierra Leone	2008, 2013	97.6, 99.3
South Africa	1998	97.0
Swaziland	2006	95.2
Tanzania	2004, 2010	98.8, 98.8
Togo	1998	98.6
Uganda	2001, 2006, 2011	95.8, 95.3, 97.5
Zambia	2002, 2007	98.2, 97.8
Zimbabwe	2006, 2011	95.0, 96.0

**Table 4:** Coefficients of country dummies used in Model 1 and Model 2

	Model 1			Model 2		
	β	SE	Exp(β)	β	SE	Exp(β)
Benin	0.229	0.173	1.257	0.256	0.173	1.292
Burkina Faso	−0.579	0.186	0.561	−0.565	0.187	0.569
Cote d’Ivoire	−0.815	0.174	0.443	−0.792	0.174	0.453
Cameroon	1.181	0.194	3.257	1.215	0.193	3.372
Congo DR	−0.182	0.140	0.834	−0.169	0.138	0.844
Congo Brazzaville	0.800	0.152	2.226	0.824	0.153	2.279
Ethiopia	−0.579	0.159	0.560	−0.585	0.160	0.557
Gabon	1.890	0.187	6.617	1.914	0.187	6.781
Ghana	0.296	0.160	1.345	0.326	0.159	1.386
Guinea	−0.077	0.218	0.926	−0.065	0.219	0.937
Kenya	1.449	0.242	4.259	1.465	0.242	4.329
Liberia	−0.452	0.179	0.636	−0.438	0.178	0.645
Lesotho	2.712	0.315	15.056	2.803	0.315	16.488
Madagascar	−0.217	0.130	0.805	−0.205	0.131	0.815
Mali	−0.719	0.189	0.487	−0.719	0.189	0.487
Mozambique	0.259	0.159	1.295	0.269	0.157	1.309
Mauritania	0.255	0.188	1.291	0.232	0.189	1.261
Malawi	1.068	0.126	2.908	1.104	0.125	3.016
Namibia	0.847	0.189	2.333	0.849	0.189	2.338
Niger	−0.774	0.194	0.461	−0.797	0.195	0.451
Nigeria	0.360	0.225	1.434	0.419	0.227	1.521
Rwanda	0.475	0.146	1.609	0.487	0.146	1.628
Senegal	−0.058	0.248	0.944	-0.058	0.249	0.944
Sierra Leone	0.284	0.166	1.328	0.307	0.166	1.359
Swaziland	0.948	0.293	2.580	0.971	0.299	2.639
Chad	−1.054	0.342	0.349	−1.062	0.340	0.346
Togo	0.943	0.233	2.567	0.979	0.232	2.661
Tanzania	0.233	0.138	1.262	0.257	0.137	1.293
Uganda	1.326	0.163	3.765	1.366	0.162	3.918
South Africa	1.582	0.255	4.866	1.573	0.255	4.819
Zambia	−0.320	0.144	0.726	−0.285	0.143	0.752
Zimbabwe	0.664	0.159	1.943	0.687	0.161	1.987
